# Osteopontin aggravates acute lung injury in influenza virus infection by promoting macrophages necroptosis

**DOI:** 10.1038/s41420-022-00904-x

**Published:** 2022-03-04

**Authors:** Jinping Wang, Xuehui Li, Yuchong Wang, Yuyu Li, Fan Shi, Hongyan Diao

**Affiliations:** 1 School of Laboratory Medicine and Life Sciences, Wenzhou Medical University, Wenzhou, China; 2 State Key Laboratory for Diagnosis & Treatment of Infectious Diseases, National Clinical Research Center for Infectious Disease, Collaborative Innovation Center for Diagnosis & Treatment of Infectious Diseases, The First Affiliated Hospital, College of Medicine, Zhejiang University, Hangzhou, China

**Keywords:** Cell death and immune response, Influenza virus

## Abstract

Infection with influenza A virus (IAV) can trigger pulmonary inflammation and lung damage. Osteopontin (OPN) is an essential regulator of cell death and immunity. However, the role and underlying mechanism of OPN in cell death in IAV-induced pulmonary injury remain poorly understood. Here, we demonstrated that OPN-deficient (OPN^−/−^) mice were insensitive to IAV, exhibiting decreased viral loads and attenuated lung injury after IAV infection compared to those in wild-type (WT) mice. Moreover, macrophage necroptosis was significantly reduced in OPN^−/−^ mice infected with IAV compared to that in infected WT mice. OPN increased the expression of necroptosis-related genes and exacerbated macrophage necroptosis in IAV-infected THP1 cells. Notably, adoptive transfer of WT bone marrow-derived macrophages (BMDMs) or OPN^−/−^ BMDMs into mice restored resistance to influenza infection, and the rescue effect of OPN^−/−^ BMDMs was better than that of WT BMDMs. Collectively, these results suggest that OPN deficiency in macrophages reduces necroptosis, which leads to a decrease in viral titers and protects against IAV infection. Therefore, OPN is a potential target for the treatment of IAV infection.

## Introduction

Influenza A virus (IAV) commonly causes respiratory disease, leading to 250,000 to 500,000 deaths annually and exerting a huge social burden worldwide. Due to frequent antigen variation and high IAV infection rate, it often causes pandemics. Although antiviral drugs make the clinical treatment of IAV infection possible, the emergence of drug-resistant mutants suggests that understanding novel targets and developing new therapeutics for IAV infections is essential. IAV is included in the *Orthomyxoviridae* family, members of which have a negative-sense, single-stranded segmented RNA genome [1, 2]. IAV infections have been reported to cause cell death in the lungs, leading to the activation of immune responses, which in turn, trigger acute respiratory disease [3, 4]. Previous studies have shown that programmed cell death pathways, especially apoptosis, play a vital role in IAV infection [5, 6]. Increasing evidence suggests that cells also undergo necroptosis, a caspase-independent cell death, after IAV entry [7–9]. Necroptosis is carried out by receptor-interacting protein kinase (RIPK) 1, RIPK3, and mixed lineage kinase domain-like protein (MLKL) [10–12]. Macrophages, innate immune cells capable of responding to IAV during the initial stage of infection, are the main immune barrier against IAV. Necroptosis in macrophages is closely related to the occurrence and development of several diseases [13–16]. For example, this process can directly lead to the formation of an atherosclerotic necrotic core and instability of plaques, thereby accelerating the process of atherosclerosis [17]. It has further been reported that necroptosis in lung macrophages is involved in the development of IAV-induced diseases [18, 19]. However, it is not clear which factors regulate necroptosis in alveolar macrophages during IAV infection. Osteopontin (OPN) is a major regulatory factor involved in cell death and immunity [20–23]. OPN plays an indispensable role in various diseases and processes, including cancer, cardiovascular diseases, and inflammation [24–26]. Research has shown that OPN regulates gene expression within the immune system [27, 28]. In addition, OPN recruits macrophages to the lesion [29]. OPN influences the state of differentiation or activation of macrophages [30, 31]. Researchers found that the activation of pro-inflammatory macrophages was effectively inhibited by OPN silencing, which subsequently alleviated the inflammatory response and disease progression [30]. Moreover, OPN-induced M2-like polarization of macrophages promotes tumor growth and pathogen proliferation [32]. Furthermore, OPN can regulate the phagocytosis of macrophages [33, 34]. The literature data corroborated that FITC-dextran phagocytized by BMDM is significantly increased in the presence of OPN [33]. However, the role of OPN in IAV-induced macrophage necroptosis remains largely elusive and requires further study. Here, we define for the first time the contribution of necroptosis-related signaling to IAV-induced pneumonia using blood samples from IAV patients and healthy controls. Furthermore, we demonstrated that IAV-infected OPN^−/−^ mice exhibited considerably decreased macrophage necroptosis in the lungs compared to that in IAV-infected WT mice. OPN^−/−^ mice infected with IAV also showed decreased IAV replication in the lungs. Moreover, OPN knockdown decreased IAV replication by reducing virus-induced necroptosis in THP1 cells. Hence, studying the role of OPN in macrophage necroptosis is important in understanding the molecular mechanism of the occurrence and development of IAV-induced lung injury and exploring clinical therapeutic targets.

## Results

### Positive correlation between the expression of OPN and necroptosis-related genes in IAV patients

**Fig. 1:**
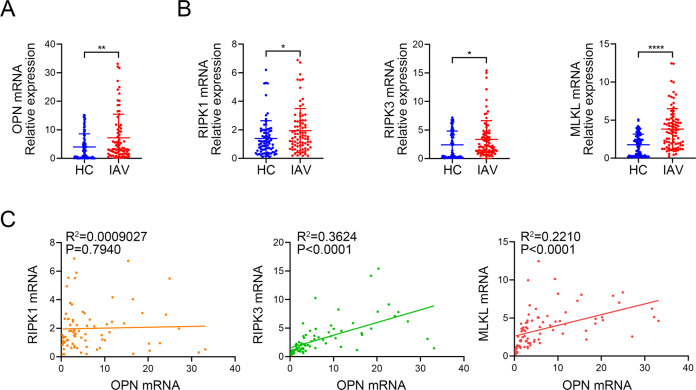
Positive correlation between Osteopontin (OPN) levels and the levels of necroptosis-related genes in influenza A virus (IAV) patients. **A** OPN mRNA expression in blood samples from healthy control (HC, *n* = 82) and IAV patients (IAV, *n* = 85). **B** RIPK1, RIPK3, and MLKL mRNA expression in blood samples from healthy control and IAV patients. **C** Spearman correlation analysis of RIPK1, RIPK3, or MLKL mRNA levels with OPN mRNA levels. Data was analyzed by Student’s *t* test (two-tailed) and expressed as mean ± SEM. Spearman rank correlation was used to test for correlations.*p < 0.05; **p < 0.01; ****p < 0.0001.

Previous studies have established that OPN is closely related to the replication of hepatitis C virus, dengue virus, and HIV and progression of the subsequent diseases [35–37], but the relationship between OPN and IAV pathogenicity has not yet been clarified. Here, we collected samples from 82 healthy individuals and 85 IAV patients and performed real-time quantitative PCR (qPCR) analysis. The data showed that OPN mRNA levels were significantly elevated in the blood samples of patients with IAV infection compared to those in the healthy control group (Fig. 1A). Necroptosis, a new type of programmed cell death, has been found to be involved in multiple tissue injuries [38–40]. Many studies have shown that Z-DNA binding protein 1 recognizes IAV RNA and activates RIPK3 [41], but whether OPN participates in necroptosis in IAV patients has not been reported. Here, we examined the mRNA levels of necroptosis-related genes in peripheral blood and observed a significant increase in RIPK1, RIPK3, and MLKL expression in IAV patients compared to that in healthy controls (Fig. 1B). In addition, we observed a strong positive association between OPN mRNA levels and RIPK3 or MLKL mRNA levels, but not with RIPK1 mRNA levels (Fig. 1C). Together, these observations suggest that OPN controls the activation of necroptosis in IAV patients.

### OPN-deficient mice demonstrated moderate lung inflammation and injury after IAV infection

**Fig. 2:**
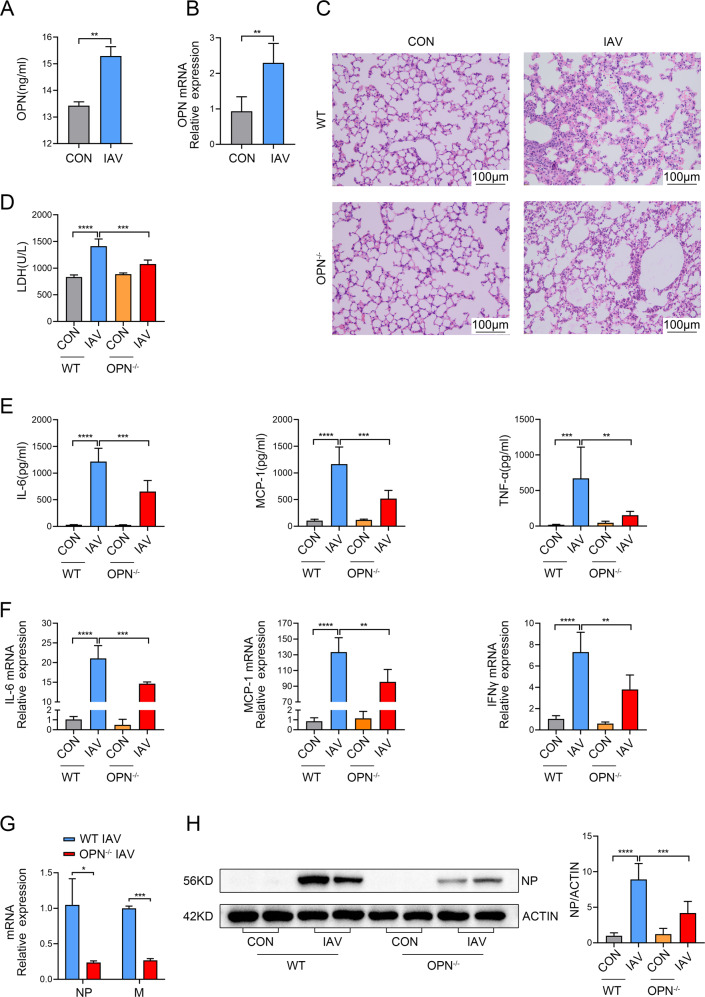
OPN deficiency reduces virus replication and decreases IAV-induced lung inflammation. **A**, **B** WT mice were infected with PR8 (1.5 × 10^3^ PFU/animal). Analysis was performed on data collected 24 h post infection. **A** OPN protein levels in BALF supernatants. **B** OPN mRNA levels in the lung homogenates. **C**–**H** WT mice or OPN^−/−^ mice were infected with PR8 (1.5 × 10^3^ PFU/animal). Analysis was performed on data collected 24 h post infection. **C** Serial lung sections of representative lungs stained with hematoxylin and eosin, scale bar = 100 μm. **D** The level of tissue injury was quantified from the LDH levels in serum. **E** BALF was obtained and inflammatory factor (IL-6, MCP-1, and TNF-α) levels in the BALF supernatant were measured using ELISA. **F** Levels of inflammatory factors (IL-6, MCP-1, and IFN-γ) in lung homogenates were measured using qPCR. **G** Total viral titers in the lung homogenates were quantified using qPCR. **H** Lung homogenates were collected and subjected to western blot analysis. Each lane corresponds to an individual mouse. Lane-loading differences were normalized by levels of ACTIN. Data was analyzed by Student’s *t* test (two-tailed) (**A**, **B**, **G**) or one-way ANOVA (**D**, **E**, **F**, **H**) and expressed as mean ± SEM. *n* = 3-7/group. *p < 0.05; **p < 0.01; ***p < 0.001; ****p < 0.0001.

C57BL/6 mice were intranasally infected with IAV PR8 strain to establish an IAV-infected mouse model. We detected a significant upregulation of OPN protein in the broncho-alveolar lavage fluid (BALF) supernatants of WT mice following IAV infection (Fig. 2A). A similar phenotype was observed for OPN mRNA expression: expression was elevated in the lung homogenates at 24 h post infection (Fig. 2B). Similar to that in IAV patients, OPN expression was also upregulated in IAV-infected mice. We next used OPN-deficient (OPN^−/−^) mice to investigate the effect of OPN on IAV infection. After WT mice and OPN^−/−^ mice were infected with IAV, lung tissues were obtained, and tissue sections were prepared for hematoxylin and eosin staining. Histological analyses of infected lungs revealed that fewer alveolar regions were injured in OPN^−/−^ mice compared to those in infected WT mice 24 h post-infection. Furthermore, infected OPN^−/−^ mice showed fewer lung-infiltrating cells and a lighter thickening of the respiratory membrane than those in the infected WT mice (Fig. 2C). As expected, OPN^−/−^ mice had lower serum lactate dehydrogenase (LDH) levels 24 h post-infection than that in WT mice (Fig. 2D). In addition, we also detected the protein expression of pro-inflammatory factors in the BALF of mice 24 h after IAV infection. The levels of interleukin-6 (IL-6), monocyte chemotactic protein-1 (MCP-1), and Tumor Necrosis Factor-α (TNF-α) in BALF were all increased after IAV infection, but the increase in OPN^−/−^ mice was less obvious than that in WT mice (Fig. 2E). Similarly, the mRNA levels of IL-6, MCP-1, and interferon-γ (IFN-γ) in the lung homogenates were lower in IAV-infected OPN^−/−^ mice than in IAV-infected WT mice (Fig. 2F). These findings suggest that OPN aggravates pathological lung damage and inflammation induced by IAV infection. Next, we tested whether the decreased inflammation in mice with deficient OPN expression was associated with increased control of viral replication during IAV infection. As shown in Fig. 2G, the transcript levels of influenza nucleoprotein (NP) and matrix protein (M) in OPN^−/−^ mice after infection were significantly reduced compared to those in WT mice. We assessed the protein expression of NP in whole-lung tissues of mice after 24 h of infection using western blotting, and the results showed that the trend was similar to that of mRNA levels (Fig. 2H). These findings demonstrate that OPN deficiency contributes to decreased viral titers, reduced inflammation, and improved lung injury.

### OPN promotes IAV-induced macrophages necroptosis in the lung

**Fig. 3:**
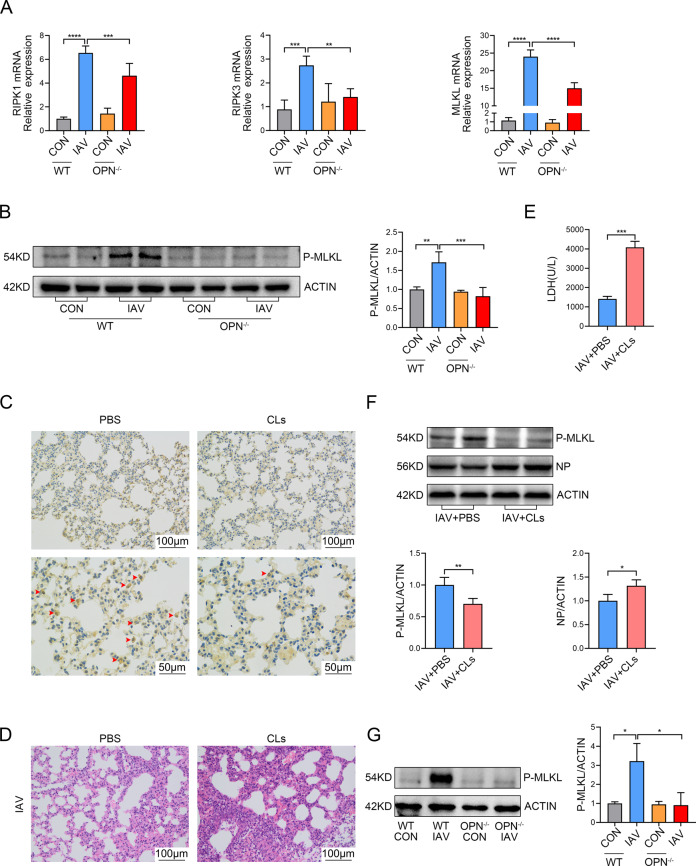
OPN promotes IAV-induced macrophage necroptosis in the lung. **A**, **B** WT and OPN^−/−^ mice were infected with PR8 (1.5 × 10^3^ PFU/animal). Analysis was performed on data collected 24 h post infection. **A** mRNA levels of necroptosis-related genes (RIPK1, RIPK3, and MLKL) in lung homogenates were measured using qPCR. **B** P-MLKL levels in the lung homogenates were measured using western blot analysis. Each lane corresponds to an individual mouse. Lane-loading differences were normalized by levels of ACTIN. **C**–**F** WT mice were intraperitoneally administered clodronate liposomes (CLs) or the same volume of sterile PBS for 24 h. **C** Serial lung sections of representative lungs stained by immunohistochemistry, scale bar = 50 μm (above) or 100 μm (below). Red arrows indicate F4/80 positive cells. **D** Serial lung sections of representative lungs of mice 24 h after PR8 (1.5 × 10^3^ PFU/animal) infection were stained with hematoxylin and eosin, scale bar = 100 μm. **E** Serum LDH levels were measured 24 h after PR8 (1.5 × 10^3^ PFU/animal) infection. **F** Lung homogenates were collected 24 h after PR8 (1.5 × 10^3^ PFU/animal) infection and subjected to western blot analysis. Each lane corresponds to an individual mouse. Lane-loading differences were normalized by levels of ACTIN. **G** WT mice or OPN^−/−^ mice were infected with PR8 (1.5 × 10^3^ PFU/animal). Macrophages in BALF were collected 24 h post infection and subjected to western blot analysis. Each lane corresponds to an individual mouse. Lane-loading differences were normalized by levels of ACTIN. Data was analyzed by Student’s *t* test (two-tailed) (**E**, **F**) or one-way ANOVA (**A**, **B**, **G**) and expressed as mean ± SEM. *n* = 3-7/group. **p < 0.01; ***p < 0.001; ****p < 0.0001.

qPCR was used to detect the expression levels of necroptosis-related genes in mouse lung tissues. The results suggest that RIPK1, RIPK3, and MLKL mRNA levels in the lung tissue samples from IAV-infected OPN^−/−^ mice were significantly lower than those in IAV-infected WT mice (Fig. 3A). Moreover, western blot analysis indicated that at 24 h post infection, OPN^−/−^ mice showed lower P-MLKL expression than that in WT mice (Fig. 3B). These findings showed that the upregulation of necroptosis-related factors induced by IAV infection is regulated by OPN levels, similar to that observed in IAV patients. Macrophages are critical for early control of viral replication [42]. To evaluate whether macrophages play an important role in IAV infection, we administered clodronate liposomes intraperitoneally to WT mice to eliminate macrophages in vivo (Fig. 3C). Histological results revealed that lung injury regions in macrophage-depleted mice were significantly increased compared to those in non-depleted mice 24 h post infection (Fig. 3D). Serum LDH levels were also markedly higher in macrophage-depleted mice after infection than in non-depleted mice (Fig. 3E). To investigate whether aggravated lung damage in infected macrophage-depleted mice was related to virus replication, we measured the expression level of influenza NP. Western blot results showed that NP levels were higher in IAV-infected macrophage-depleted mice than in IAV-infected non-depleted mice (Fig. 3F). These results suggest that macrophages play an important role in viral clearance during IAV infection. More importantly, IAV-induced necroptosis was considerably reduced after macrophage clearance, indicating that most of the cells undergoing necroptosis in the lung were macrophages (Fig. 3F). Previous studies have reported that cell death is one of the most common causes of decreased cell numbers [43, 44]. Moreover, we found that OPN regulates necroptosis and viral replication (Figs. 1C and 3A, B). Therefore, we explored whether OPN regulates macrophage necroptosis to control virus clearance. We obtained alveolar macrophages from the BALF of the mice in the four groups and performed western blot analysis. The P-MLKL level was significantly decreased in alveolar macrophages from IAV-infected OPN^−/−^ mice compared to that in IAV-infected WT mice (Fig. 3G). Taken together, these findings strongly suggest that IAV infection triggers macrophage necroptosis and that OPN deficiency attenuates IAV-induced macrophage necroptosis to restrict IAV replication and lung damage.

### OPN promotes IAV replication by enhancing IAV-induced necroptosis in macrophages in vitro

**Fig. 4:**
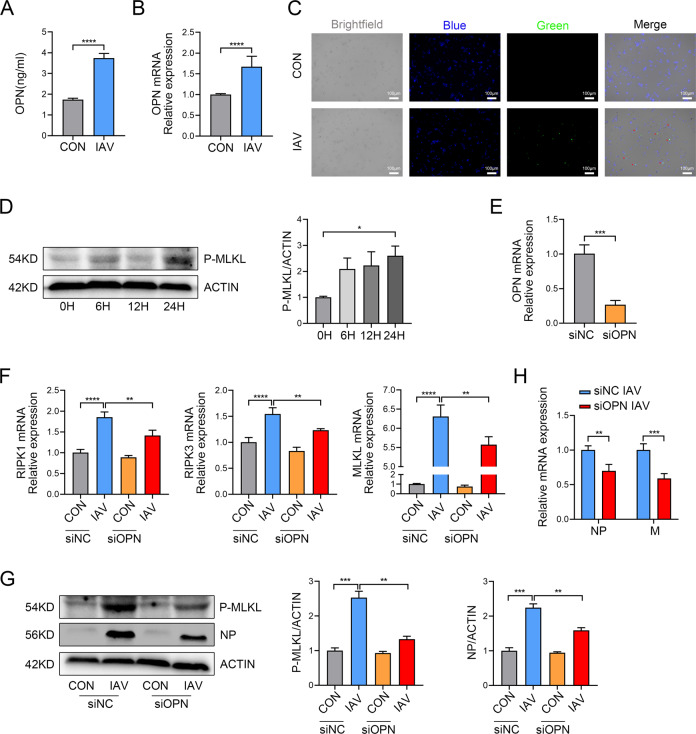
OPN knockdown inhibits necroptosis in THP1 cells infected with IAV. PMA was added to the THP1 culture for 24 h to stimulate THP1 cells adherence and differentiation into macrophage-like cells. **A**, **B** THP1 cells were infected with 3 MOI PR8 for 24 h. **A** OPN protein levels in the cell culture supernatant. **B** OPN mRNA levels in the cell lysate. **C** THP1 cells were infected with GFP-PR8 (green) for 24 h and stained with DAPI (blue). Red arrows indicate positive cells. **D** THP1 cells were infected with 3 MOI PR8 for 0, 6, 12, and 24 h. Cell lysates were collected and subjected to western blot analysis. Each lane corresponds to an individual mouse. Lane-loading differences were normalized by levels of ACTIN. **E**–**G** OPN expression in THP1 cells was silenced using OPN-specific small interfering RNA (siRNA) or nonspecific siRNA (siNC). THP1 cells were infected with 3 MOI PR8 for 24 h. **E** OPN gene silencing efficiency in THP1 cells. **F** RIPK1, RIPK3, and MLKL mRNA levels in THP1 cells. **G** Cell lysates were collected and subjected to western blot analysis. Each lane corresponds to an individual mouse. Lane-loading differences were normalized by levels of ACTIN. **H** Total viral titers in cell lysates were quantified by qPCR. Data was analyzed by Student’s *t* test (two-tailed) (**A**, **B**, **E**, **H**) or one-way ANOVA (**D**, **F**, **G**) and expressed as mean ± SEM. *n* = 3-7/group. **p < 0.01; ***p < 0.001; ****p < 0.0001.

We infected THP1 cells with IAV and extracted cellular RNA or protein to detect the OPN expression level. The results showed that THP1 cells secreted OPN without stimulation, and OPN increased remarkably at the mRNA and protein levels after IAV infection (Fig. 4A, B). Next, we infected THP1 cells with recombinant PR8 expressing GFP and found that THP1 cells could phagocytize viruses (Fig. 4C). We subsequently demonstrated that IAV induced necroptosis-related gene expression in macrophages. IAV infection of THP1 cells resulted in an increase in RIPK1, RIPK3, and MLKL mRNA levels and P-MLKL protein levels in a time-dependent manner most significantly at 24 h (Fig. S1 and Fig. 4D). To further explore the role of OPN in macrophage necroptosis, we silenced OPN-associated genes in THP1 cells using OPN-specific small interfering RNA (siRNA), with a silencing efficiency of approximately 75% (Fig. 4E). The knockdown of OPN in THP1 cells markedly decreased necroptosis-related molecules at the gene expression and protein levels after IAV infection compared to those with control siRNA (Fig. 4F, G). These results indicated that OPN promoted IAV-induced macrophage necroptosis. Similarly, OPN knockdown macrophages infected with IAV exhibited significantly lower viral loads than those in control macrophages infected with IAV (Fig. 4G, H). Collectively, these findings were validated when macrophages were treated with OPN-specific siRNA, further implicating the role of OPN in promoting macrophage necroptosis during IAV infection.

### Adoptive transfer of OPN^−/−^ bone marrow-derived macrophages (BMDMs) protected mice against IAV infection more than WT BMDMs

**Fig. 5:**
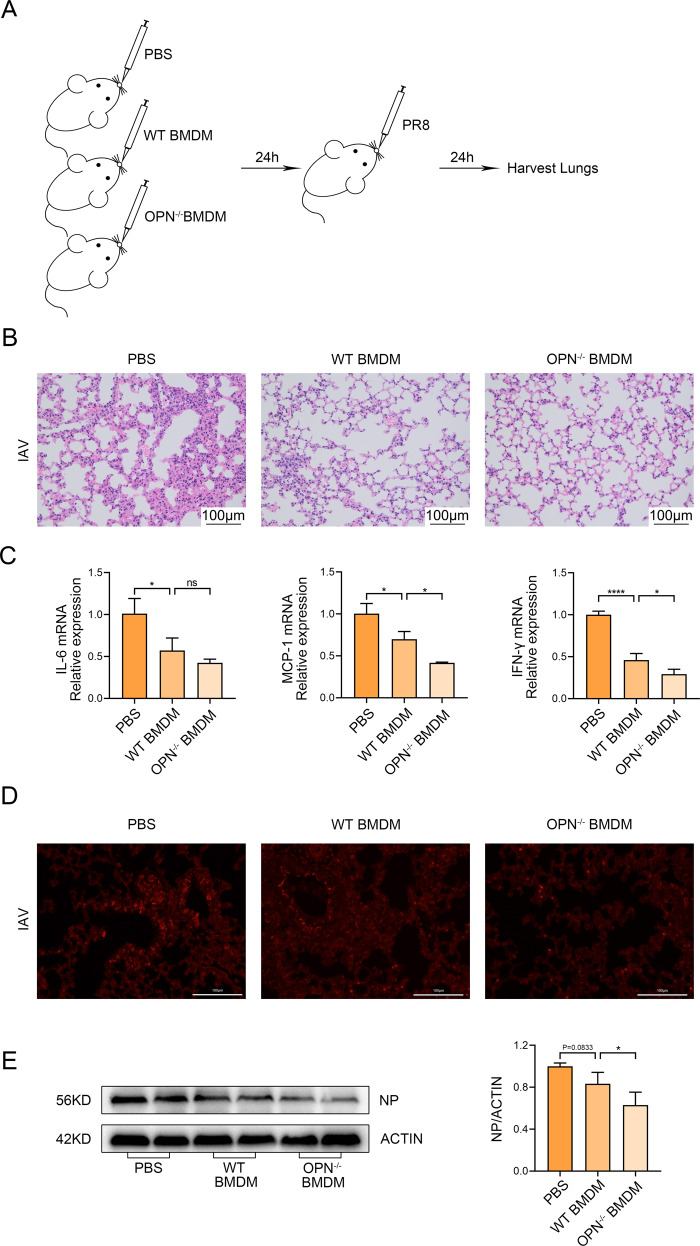
Adoptive transfer of OPN^−/−^ BMDM reduces tissue injury in mice induced by subsequent PR8 infection. **A**–**E** WT or OPN^−/−^ BMDMs were intranasally transferred to WT recipient mice. One day after transfer, mice were infected with PR8 (1.5 × 10^3^ PFU/animal) and lung tissues were collected 24 h after infection. **A** Diagrammatic representation of adoptive transfer. **B** Serial lung sections of representative lungs stained with hematoxylin and eosin, scale bar = 100 μm. **C** Inflammatory factor (IL-6, MCP-1, and IFN-γ) levels in lung homogenates were measured using qPCR. **D** Representative images of immunofluorescence staining for P-MLKL in the lung. **E** Lung homogenates were collected and subjected to western blot analysis. Each lane corresponds to an individual mouse. Lane-loading differences were normalized by levels of ACTIN. Data was analyzed by one-way ANOVA and expressed as mean ± SEM. *n* = 3-4/group. *p < 0.05; ****p < 0.0001; ns denote no statistical significance.

To further emphasize the crucial role of OPN in macrophage necroptosis, we obtained BMDMs from mice and performed adoptive transfer of macrophages. Bone marrow-derived cells from WT mice or OPN^−/−^ mice were isolated and stimulated to differentiate into BMDMs by administering granulocyte-macrophage colony stimulating factor. Subsequently, we transferred WT or OPN^−/−^ BMDMs intranasally into WT recipient mice. One day after transfer, macrophages were colonized in the lungs of mice, and the mice were then challenged with IAV. One day post-infection, lung tissues of mice in the different groups were obtained (Fig. 5A). As shown in Fig. 5B, the adoptive transfer of BMDMs restored the resistance of mice to IAV, and milder inflammation and tissue damage was seen in mice that received OPN^−/−^ BMDMs than in the mice that received WT BMDMs. To further evaluate whether the transfer of BMDMs affected IAV-induced pulmonary inflammation, qPCR was used to assess the mRNA expression of pro-inflammatory factors in whole-lung tissues of mice. Transcript levels of IL-6, MCP-1, and IFN-γ in mice that received OPN^−/−^ BMDMs decreased after IAV infection compared to those in mice that received WT BMDMs (Fig. 5C). We performed immunofluorescent staining for P-MLKL on lung tissues and observed that the staining intensity of P-MLKL was significantly lower in mice receiving OPN^−/−^ BMDMs than in mice receiving WT BMDMs (Fig. 5D). Moreover, the viral NP level in mice receiving OPN^−/−^ BMDMs was lower than that in mice receiving WT BMDMs, which may depend on the resistance of OPN^−/−^ BMDMs to necroptosis (Fig. 5E). Thus, these results establish that macrophages play an indispensable role in IAV infection and that OPN^−/−^ BMDMs can better protect against IAV infection than WT BMDMs. In conclusion, our data demonstrate that IAV induces the expression of OPN, which promotes IAV-induced macrophage necroptosis to increase IAV replication and lung damage.

## Discussion

In this study, we aimed to determine whether OPN regulates IAV-induced necroptosis. We found that OPN-deficient macrophages alleviate lung inflammation and injury by inhibiting necroptosis during IAV infection. The knockdown of OPN expression in THP1 cells further decreased necroptosis and reduced virus copies after IAV infection. To the best of our knowledge, this is the first direct evidence of the role of OPN in IAV-induced necroptosis.

Recent studies have reported that IAV induces necroptosis in IAV-infected mouse models. However, changes in necroptosis in patients with IAV have not been investigated. Here, we analyzed the expression levels of necroptosis-related genes in blood samples from healthy individuals and IAV patients and found that RIPK1, RIPK3, and MLKL mRNA levels in IAV patients were higher than those in the control group. Moreover, we demonstrated that OPN levels were positively correlated with RIPK3 and MLKL levels. These data suggest that OPN may have a beneficial effect on necroptosis during clinical IAV infection. OPN^−/−^ mice were used to determine the role of OPN in IAV-induced lung injury. The results showed that IAV-infected OPN^−/−^ mice had lower inflammatory factor expression and less viral replication than in IAV-infected WT mice. Our data establish that OPN deficiency protects mice from IAV-induced pneumonia. In addition, we found that RIPK1, RIPK3, and MLKL mRNA levels and P-MLKL protein levels in the lung tissues of IAV-infected OPN^−/−^ mice were decreased compared to those in IAV-infected WT mice, further emphasizing that OPN regulates necroptosis after IAV infection.

The role of macrophages in IAV infection remains unclear. Although macrophages seem to play a crucial role in the early antiviral response against IAV infection [45, 46], some studies have shown that macrophages significantly contribute to alveolar epithelial cell (AEC) apoptosis through the release of TNF-related apoptosis-inducing ligands during IAV infection [6]. Our findings strongly suggest that macrophages promote virus clearance in the early stages of IAV infection. These results were consistently emphasized by increased viral loads, aggravated lung damage, and inflammation in macrophage-depleted mice. Moreover, the adoptive transfer of BMDMs restored the resistance of mice to IAV. Therefore, it is reasonable to believe that macrophages may be the first immune cells in the lungs to encounter IAV, acting as guardians in the early stages of infection. Notably, the activation of IAV-induced P-MLKL decreased significantly after macrophage depletion, indicating that macrophages accounted for most of the P-MLKL expression after infection, helping them to undergo necroptosis. We further determined that macrophage necroptosis increased significantly after IAV infection by obtaining purified pulmonary macrophages and detecting P-MLKL levels. In vitro necroptosis of THP1 cells was also examined.

The role of necroptosis in the occurrence and development of viral infectious diseases is controversial. Although some studies claim that necroptosis participates in virus clearance [8], other studies have suggested that it also leads to lung injury [47]. Our results suggest that macrophage necroptosis aggravates IAV-induced lung injury. Given our observation on the participation of OPN during IAV infection of macrophages, we sought to elucidate the role of OPN in IAV infection. In the THP1 cells, OPN knockdown decreased the mRNA expression of RIPK1, RIPK3, and MLKL, as well as the protein level of P-MLKL, after IAV infection. In addition, mice that received OPN^−/−^ BMDMs had less lung damage than that in the mice that received WT BMDMs, showing that OPN^−/−^ BMDMs were not sensitive to necroptosis. The in vivo and in vitro results indicate that OPN reduces virus clearance and aggravates lung inflammation by inducing macrophage necroptosis.

Previous studies have shown that necroptosis of AECs prevents infected cells from becoming “factories” for virus replication [8, 48]. In this study, we detected the expression of necroptosis-related genes in whole-lung and alveolar macrophages but did not examine the effect of necroptosis of AECs on virus replication. Therefore, further studies should isolate AECs and alveolar macrophages to detect the expression level of necroptosis-related molecules and more strictly exclude the influence of AECs. In addition, to be more certain that the results obtained in our in vivo studies depended on necroptosis, MLKL knockout mice or MLKL inhibitors should be used to inhibit necroptosis. In summary, our data indicated that OPN regulates macrophage necroptosis. However, further investigation is required to elucidate the underlying mechanisms.

In conclusion, our study reveals a novel mechanism by which OPN promotes macrophage necroptosis, contributing to increased lung injury and inflammation during IAV infection. Regulation of OPN expression may, therefore, be a new strategy for the treatment of IAV patients.

## Materials/subjects and methods

### Human samples

Human blood samples from IAV infected patients and healthy controls were obtained from donors with their informed consent. From 2018 to 2019, 85 IAV infected patients and 82 healthy controls were recruited from the First Affiliated Hospital, College of Medicine, Zhejiang University. All patients were confirmed to be IAV-positive by detecting throat swabs with specific probes and primers.

### Animal experiments

Mice were housed under specific pathogen-free conditions in the animal facility of The First Affiliated Hospital, College of Medicine, Zhejiang University and all experiments on animals were approved by the Animal Ethics Committee of The First Affiliated Hospital, College of Medicine, Zhejiang University. 6–8 weeks old C57BL/6 mice were purchased from Hangzhou Ziyuan Laboratory Animal CO. LTD. OPN^−/−^ mice provided from The Jackson Laboratory were bred at the animal facility of Zijingang Campus of Zhejiang University. Experiments were performed using age- and sex-matched mice.

### Isolation of alveolar macrophages and cell lines

After mice were euthanized, the trachea was exposed, cannulated, and BALF was collected by injecting and pooling 1 ml sterile PBS five times. The cells of BALF were cultured in Dulbecco’s modified Eagle’s medium (DMEM, Sigma–Aldrich, Merck KGaA, Darmstadt, Germany) containing 10% fetal bovine serum (FBS, Gibco, Gaithersburg, MD, USA) and 100 U/mL penicillin/streptomycin (Sigma–Aldrich). After 2 h adhesion, alveolar macrophages were washed with PBS and treated with lysis buffer. Human monocyte cell line THP1 were obtained from the State Key Laboratory for Diagnosis and Treatment of Infectious Disease, cultured in 1640 medium (Sigma–Aldrich) supplemented with 10% FBS and 100 U/mL penicillin/streptomycin, and plated in 24-well plates at a density of 4-5× 10^5^. Phorbol-12-myristate-13-acetate (PMA; Sigma–Aldrich; 100 μg/ml) was added to the THP1 culture for 24 h to stimulate THP1 cells adherence and differentiation into macrophage-like cells.

### Reagents

HiScript II QRT SuperMix for qPCR (Vazyme, Nanjing, China), ChamQ Universal SYBR qPCR Master Mix (Vazyme, Nanjing), the lactate dehydrogenase (LDH) cytotoxicity assay kit (FUJIFILM, China). The following primary antibodies were used: Anti-mouse P-MLKL (1:1000, Abcam, Cambridge, MA, USA, ab196436), Anti-β-actin (1:1000, GenScript Biotechnology, Nanjing, China, A00702), Anti-human P-MLKL (1:1000, Cell Signaling Technology, Danvers, MA, USA, 91689 S), Influenza A Virus NP antibody (1:5000, GeneTex, Irvine, CA, USA, GTX125989). HRP anti-rabbit secondary antibody (1:8000, HuaAn Biotechnology, China, HA1001), HRP anti-mouse secondary antibody (1:8000, HuaAn Biotechnology, HA1006), Halt protease and phosphatase inhibitor cocktail (Selleck, USA), Trizol reagent (Takara, Kyoto, Japan), RIPA Lysis Buffer (Beyotime Biotechnology, China). Mouse IL-6 enzyme-linked immunosorbent assay (ELISA) kit (Invitrogen, Carlsbad, CA, USA), Mouse MCP-1 ELISA kit (Invitrogen), Mouse TNF-α ELISA kit (eBioscience, San Diego, CA, USA), Mouse OPN ELISA kit (Multi Sciences, China), Human OPN ELISA kit (Multi Sciences), Clodronate liposomes (YEASEN, China).

### Viruses and infections

Influenza A virus (Puerto Rico/8/1981 H1N1 (PR8)) were kindly provided by Professor Wang from Chinese Academy of Medical Sciences & Peking Union Medical College. IAV strains were propagated by allantoic inoculation of 10-day-old specific pathogen-free embryonated hen’s eggs at 38 °C for 72 h. Allantoic fluid was collected and HA activity was tested with 0.5% chicken erythrocytes. Viruses were titrated using standard plaque assay in Madin-Darby canine kidney (MDCK) cells and viral stock was stored at −80 °C. 6–8 weeks old mice, randomly divided into different groups, were anesthetized by IP injection of pentobarbital sodium (50 mg/kg), then administered either intranasal instillation of 50 µl sterile saline control vehicle or 50 µl saline containing 1.5 × 10^3^ plaque-forming units (PFU) PR8. For cell culture experiments, OPN-specific siRNA or nonspecific siRNA was transfected into adherent THP1 cells using Lipofectamine 2000 (Invitrogen) according to the manufacturer’s instructions. Then, near-confluent monolayers of cells were infected with virus (MOI = 3) in DMEM containing 2% FBS for 1 h in a humidified tissue culture incubator maintained at 37 °C and 5% CO_2_. Following infection, the inoculum was removed and replaced with growth medium.

### mRNA isolation and quantitative PCR (qPCR) analysis

Total RNA was isolated and purified using Trizol reagent. Reverse transcription was performed on 5 μg total RNA in a final volume of 10 μl using reverse transcriptase and random primers. qPCR was performed using 1 μl of cDNA, 5 μl of SYBR Green and 10 μM of each primer in a total volume of 10 μl. The parameters of the thermal cycling were 95 °C for 30 s, followed by 40 cycles at 95 °C for 10 s and 60 °C for 30 s, finally 95 °C for 15 s, 60 °C for 60 s and 95 °C for 15 s. The primer sequences were as follows: m-GAPDH-F, 5′-AGGTCGGTGTGAACGGATTTG-3′ and m-GAPDH-R, 5′-GGGGTCGTTGATGGCAACA-3′; h-GAPDH-F, 5′-GGAGCGAGATCCCTCCAAAAT-3′ and h-GAPDH-R, 5′-GGCTGTTGTCATACTTCTCATGG-3′; m-OPN-F, 5′-ATCTCACCATTCGGATGAGTCT-3′ and m-OPN-F, 5′-TGTAGGGACGATTGGAGTGAAA-3′; h-OPN-F, 5′-CTCCATTGACTCGAACGACTC-3′ and h-OPN-R, 5′-CAGGTCTGCGAAACTTCTTAGAT-3′; m-RIPK1-F, 5′-GACAGACCTAGACAGCGGAG-3′ and m-RIPK1-R, 5′-CCAGTAGCTTCACCACTCGAC-3′; m-RIPK3-F, 5′-CGCATCTGCTCAACGACGA-3′ and m-RIPK3-R, 5′-TCGCTGCCATTTCCGTGAC-3′; m-MLKL-F, 5′-TATGTCTCCCCTGAGAGACTGAAAA-3′ and m-MLKL-R, 5′-TTCCCAGAGTACAATTCCAAAGCTA-3′; m-IL-6-F, 5′-CTGCAAGAGACTTCCATCCAG-3′ and m-IL-6-R, 5′-AGTGGTATAGACAGGTCTGTTGG-3′; m-MCP-1-F, 5′-TAAAAACCTGGATCGGAACCAAA-3′ and m-MCP-1-R, 5′-GCATTAGCTTCAGATTTACGGGT-3′; m-IFN-γ-F, 5′-GCCACGGCACAGTCATTGA-3′ and m-IFN-γ-R, 5′-TGCTGATGGCCTGATTGTCTT-3′; h-RIPK1-F, 5′-GGGAAGGTGTCTCTGTGTTTC-3′ and h-RIPK1-R, 5′-CCTCGTTGTGCTCAATGCAG-3′; h-RIPK3-F, 5′-ATGTCGTGCGTCAAGTTATGG-3′ and h-RIPK3-R, 5′-CGTAGCCCCACTTCCTATGTTG-3′; h-MLKL-F, 5′-AGGAGGCTAATGGGGAGATAGA-3′ and h-MLKL-R, 5′-TGGCTTGCTGTTAGAAACCTG-3′; IAV M-F, 5′-GACCAATCCTGTCACCTCTGAC-3′ and IAV M-R 5′-GGGCATTTTGGACAAAGCGTCTACG-3′; IAV NP-F, 5 ′-AATAAGGCGAATCTGGCGCCAA-3′ and IAV NP-R,5 ′-CATCCTGGGATCCATTCCGGT-3′.

### Western blotting

Lungs and cells were lysed in RIPA lysis buffer supplemented with cocktails. The extracted protein was resolved by SDS-PAGE and transferred onto PVDF membranes. The membrane was blocked at room temperature for 1 h and incubated with primary antibody overnight at 4 °C. Primary antibodies were followed by HRP-conjugated secondary antibodies and signal was detected using Western ECL substrate. Densitometry analyses were performed using ImageJ software.

### Quantification of OPN and cytokines by ELISA

BALF was collected in 500 μl sterile PBS and centrifuged at 1000 rpm at 4 °C for 5 min to remove cells. ELISA plates were coated with capture antibody in coating buffer overnight at 4 °C. Plates were washed 3 times with wash buffer and blocked with ELISA diluent at room temperature for 1 h. Plates were washed at least once with wash buffer, and samples were added to wells and incubated at room temperature for 2 h. Plates were washed 5 times and incubated with detection antibody at room temperature for 1 h. Plates were washed 5 times, and streptavidin-HRP were added and incubated for 30 min at room temperature. Plates were then washed and developed using TMB solution and development was stopped with stop solution. Read plate at 450 nm and 570 nm. Subtract the values of 570 nm from those of 450 nm and analyze data. OPN protein levels in BALF supernatants and cell supernatants were measured in a similar way using human or mouse ELISA kits according to the manufacturer’s protocol.

### Histology

Mouse lung tissues were fixed with 4% paraformaldehyde (PFA) at room temperature. According to the manufacturer’s instructions, the slides were stained with hematoxylin and eosin and were observed under an optical microscope.

### LDH assay

According to the manufacturer’s instructions, the amount of LDH released in the serum was evaluated using the cytotoxicity LDH assay kit and the result was automatically recorded by the biochemical analyzer.

### Macrophage depletion

The mice were intraperitoneally administered 200 μl of Clodronate liposomes at a concentration of 5 mg/mL or the same volume of sterile PBS. Twenty-four hours later, proceed to the next experiment.

### Macrophage adoptive-transfer experiment

For BMDM, femora and tibiae of WT and OPN knockout C57BL/6 mice were flushed with PBS. Cells were cultured for 7 d in DMEM medium supplemented with 10 ng/ml recombinant mouse granulocyte-macrophage colony stimulating factor and were re-fed on day 3. Then, BMDM were counted, re-suspended and 1 × 10^6^ cells were transferred into WT recipient mice via i.n. route [49, 50]. They were infected with IAV PR8 one day later and lung specimens were collected after 24 h of infection [49].

### Statistical analysis

Statistical analyses were performed using GraphPad Prism Software and expressed as means ± Standard Error of Mean (SEM). Data were analyzed by Student’s *t* test and one-way ANOVA. Spearman rank correlation was used to test for correlations. *p* < 0.05 was defined statistically significant. Asterisks in the figures denote statistical significance (*p < 0.05, **p < 0.01, ***p < 0.001, ****p < 0.0001) and ns denote no statistical significance. Data are shown as representative of at least 3 independent experiments.

## Supplementary information

Figure S1

original western blots

## Data Availability

The data that were analyzed during the current study are available from the corresponding author on reasonable request.
