# Supplementary material for: Osteopontin aggravates acute lung injury in influenza virus infection by promoting macrophages necroptosis
Source: Cell Death Discov. 2022 Mar 4;8:97. doi: 10.1038/s41420-022-00904-x (PMC8897470; doi:10.1038/s41420-022-00904-x)
Supplement: Supplementary file 1 — Figure S1 [file 41420_2022_904_MOESM1_ESM.docx]

**
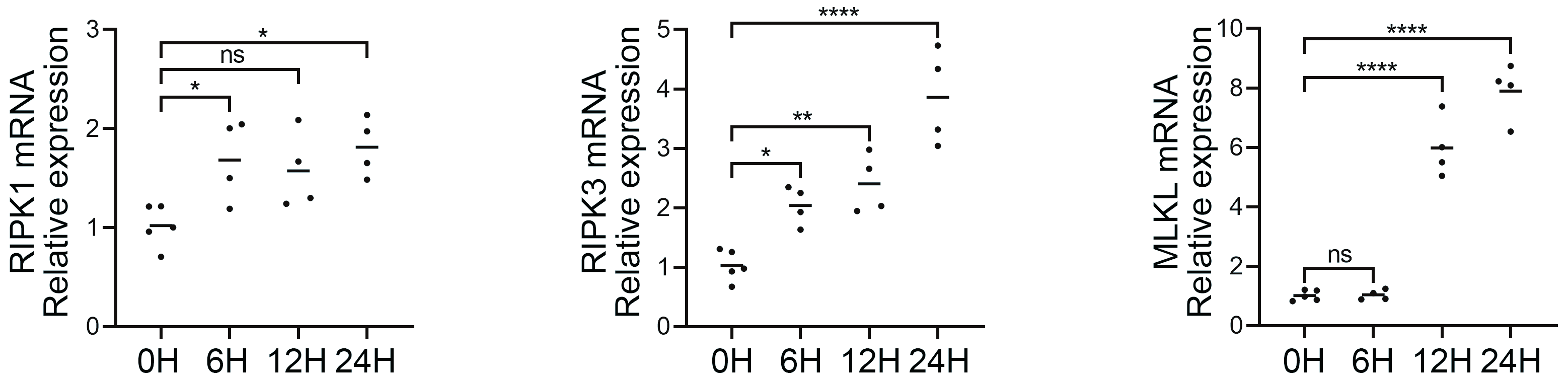
**

**Figure S1.OPN knockdown inhibits necroptosis in THP1 cells infected with IAV**

PMA was added to the THP1 culture for 24h to stimulate THP1 cells adherence and differentiation into macrophage-like cells. THP1 cells were infected with 3 MOI PR8 for 0, 6, 12, 24 hours. RIPK1, RIPK3 and MLKL mRNA level in cell lysates. Data was analyzed by one-way ANOVA and expressed as mean ± SEM. n=4-5/group. *=p<0.05; **=p<0.01; ****=p<0.0001; ns denote no statistical significance.
