# Supplementary material for: Osteopontin aggravates acute lung injury in influenza virus infection by promoting macrophages necroptosis
Source: Cell Death Discov. 2022 Mar 4;8:97. doi: 10.1038/s41420-022-00904-x (PMC8897470; doi:10.1038/s41420-022-00904-x)

Figure 2H ACTIN


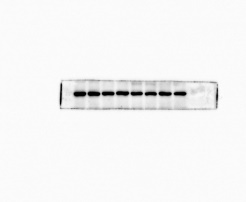


Figure 2H NP


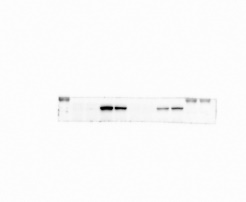


Figure 3B ACTIN


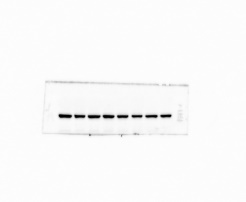


Figure 3B P-MLKL


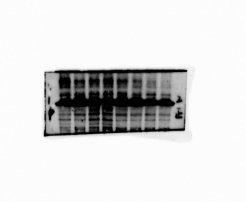


Figure 3F ACTIN


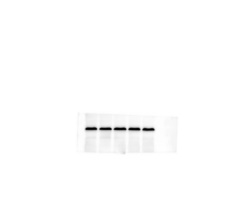


Figure 3F P-MLKL


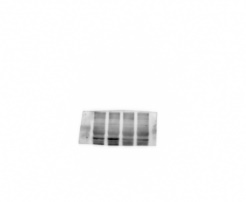


Figure 3F NP


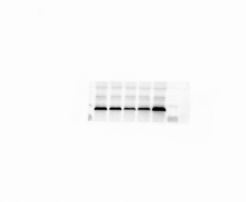


Figure 3G ACTIN


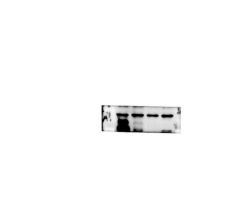


Figure 3G P-MLKL


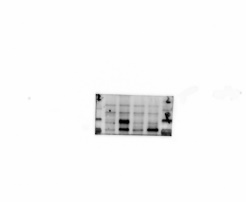


Figure 4D ACTIN


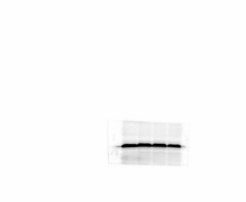


Figure 4D P-MLKL


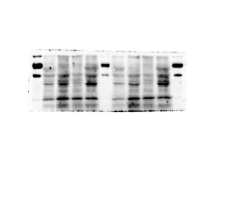


Figure 4G ACTIN


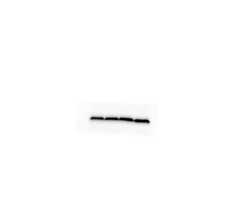


Figure 4G P-MLKL


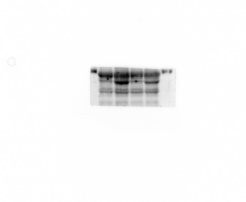


Figure 4G NP


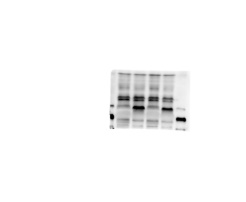


Figure 5E ACTIN


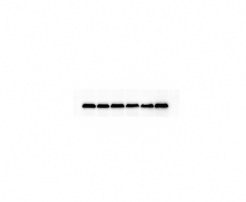


Figure 5E NP


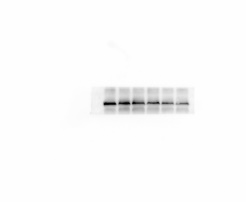

Supplement: Supplementary file 2 — original western blots [file 41420_2022_904_MOESM2_ESM.docx]
